# Metabolomics (Non-Targeted) of Induced Type 2 Diabetic Sprague Dawley Rats Comorbid with a Tissue-Dwelling Nematode Parasite

**DOI:** 10.3390/ijms242417211

**Published:** 2023-12-07

**Authors:** Innocent Siyanda Ndlovu, Selaelo Ivy Tshilwane, Philile Ignecious Ngcamphalala, Andre’ Vosloo, Mamohale Chaisi, Samson Mukaratirwa

**Affiliations:** 1 School of Life Sciences, University of KwaZulu-Natal, Westville Campus, Durban 4001, South Africa; syandandlovu111@gmail.com (I.S.N.); 212510872@stu.ukzn.ac.za (P.I.N.); vosloo@ukzn.ac.za (A.V.); 2 Department of Veterinary Tropical Diseases, Faculty of Veterinary Science, University of Pretoria, Pretoria 0110, South Africa; selaelo.tshilwane@up.ac.za (S.I.T.); m.chaisi@sanbi.org.za (M.C.); 3 Foundational Biodiversity Science, South African National Biodiversity Institute, Pretoria 0001, South Africa; 4 One Health Center for Zoonoses and Tropical Veterinary Medicine, School of Veterinary Medicine, Ross University, Basseterre KN0101, Saint Kitts and Nevis

**Keywords:** type 2 diabetes, *Trichinella zimbabwensis*, metabolomics, amino acids, carbohydrates, Sprague Dawley rats

## Abstract

Type 2 diabetes is a non-communicable metabolic syndrome that is characterized by the dysfunction of pancreatic β-cells and insulin resistance. Both animal and human studies have been conducted, demonstrating that helminth infections are associated with a decreased prevalence of type 2 diabetes mellitus (T2DM). However, there is a paucity of information on the impact that helminths have on the metabolome of the host and how the infection ameliorates T2DM or its progression. Therefore, this study aimed at using a non-targeted metabolomics approach to systematically identify differentiating metabolites from serum samples of T2DM-induced Sprague Dawley (SD) rats infected with a tissue-dwelling nematode, *Trichinella zimbabwensis*, and determine the metabolic pathways impacted during comorbidity. Forty-five male SD rats with a body weight between 160 g and 180 g were used, and these were randomly selected into control (non-diabetic and not infected with *T. zimbabwensis*) (n = 15) and T2DM rats infected with *T. zimbabwensis* (TzDM) (n = 30). The results showed metabolic separation between the two groups, where d-mannitol, d-fructose, and glucose were upregulated in the TzDM group, when compared to the control group. L-tyrosine, glycine, diglycerol, L-lysine, and L-hydroxyproline were downregulated in the TzDM group when compared to the control group. Metabolic pathways which were highly impacted in the TzDM group include biotin metabolism, carnitine synthesis, and lactose degradation. We conclude from our study that infecting T2DM rats with a tissue-dwelling nematode, *T. zimbabwensis*, causes a shift in the metabolome, causing changes in different metabolic pathways. Additionally, the infection showed the potential to regulate or improve diabetes complications by causing a decrease in the amino acid concentration that results in metabolic syndrome.

## 1. Introduction

Diabetes mellitus is among the most common non-communicable metabolic diseases that is caused by a deficiency in or diminished effectiveness of endogenous insulin [1]. The prevalence of diabetes appears to be increasing rapidly [2]. The ninth edition of the International Diabetes Federation (IDF) reported an estimated prevalence of 463 million adults in 2019 [3]. Adaptation to the Western lifestyle, diet, and genetic predisposition are reported to be the major causes of the increase in type 2 diabetes mellitus (T2DM) cases in sub-Saharan African countries [4,5,6]. Reduced arterial perfusion, suppressed immune response, and neuropathy in T2DM exacerbates the frequency and severity of infectious diseases [7]. Recently, epidemiological studies have shown that helminth infections play a significant role in the etiology of diabetes. Additionally, it has been hypothesized that a decrease in helminth infections may influence the increase in incidence of T2DM, inflammatory diseases, insulin resistance, and obesity [8].

It is estimated that approximately 1.5 billion people globally are infected with one or more parasite species [9,10]. Parasitic infections such as soil-transmitted helminths, hydatidosis, toxoplasmosis, and cysticercosis infect a large proportion of the global population, especially in developing countries [1,11], creating a scenario of comorbid cases of T2DM and helminthic infections [12]. Experimental evidence and epidemiological studies have shown that helminth infections modulate glucose levels and play a protective role against the development or progression of T2DM [13,14]. Epidemiological studies from helminth-endemic areas such as rural China, Indonesia, aboriginal communities from northwest Australia, and India reported an inverse relationship between helminth infection and the incidence of T2DM [15]. Parasite species that have been studied to have shown delays in the onset of hyperglycemia in rats include *Syphacia muris* [16]. Silas and Tshilwane [17] conducted a study on the comorbidity of *Trichinella zimbabwensis* and T2DM in Sprague Dawley rats and reported low blood glucose and increased insulin levels in animals that were diabetic and infected with *T. zimbabwensis*. However, the metabolite shifts in the animals were not studied, except for their glucose indices. The mechanism(s) of how this occurs has been an area of recent studies and postulations, with evidence that helminths and their excretory/secretory products regulate the immune responses of the mammalian host and their influence on macrophage and β-cell crosstalk [18]. Despite the focus on the modulation of immune cells and their effectors, an additional aspect on the impact of these on β-cells related to signal exchanges between β-cells and macrophages, resulting in crosstalk between macrophages and β-cells to promote their function and survival, has been advocated [18].

Diabetes is currently diagnosed via oral glucose tolerance tests, glycated hemoglobin, and fasting blood glucose [19]. With the mentioned diagnostic tools, up to 60% of diabetes cases are never captured or diagnosed [20]. The reasons behind this are due to the reduced sensitivity of assays at predicting diabetic thresholds. Metabolomics is an approach to study metabolites and metabolic changes and has been applied in both diabetes studies and parasite infections to identify predictive biomarkers to enable early interventions against type 2 diabetes and its comorbidities [21].

With the global increase in the prevalence of T2DM–helminth comorbidity in developing countries, there is a paucity of reported studies on the metabolite changes that occur during comorbidity with T2DM and helminth infections. Therefore, this study aimed to use a laboratory animal model and apply a gas chromatographic time-of-flight mass spectrometry (GCxGC-TOF-MS) metabolomics approach to determine the metabolite changes in experimentally induced T2DM Sprague Dawley rats infected with a tissue-dwelling nematode parasite, *T. zimbabwensis*, and further identify the differentiating metabolites and biochemical pathways impacted or perturbed during infection and implications for the prognosis of T2DM.

## 2. Results

### 2.1. Trichinella zimbabwensis Burden in Type 2 Diabetic Rats

**Figure 1:**
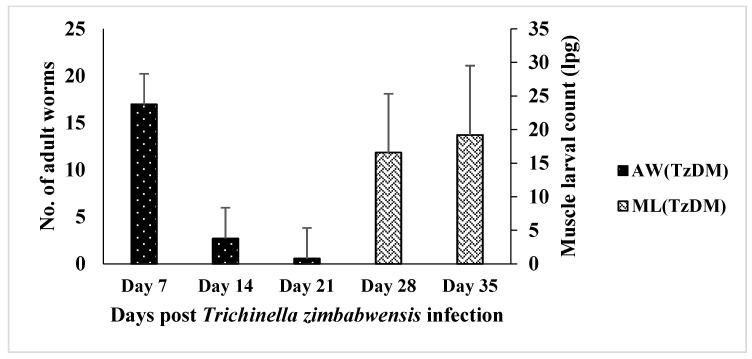
Mean number (±SEM) of *Trichinella zimbabwensis* adult worms (AW) and larvae per gram (lpg) of muscle larvae (ML) recovered from the intestines and muscles of male type 2 diabetes mellitus-induced Sprague Dawley rats. TzDM = type 2 diabetes mellitus-induced rats infected with *Trichinella zimbabwensis*.

The adult worm load in the intestine of the TzDM rats was significantly higher at day 7 pi, with a mean of 16.96 ± 3.69 lpg (Figure 1). There was a decrease in the adult worm count from day 7 pi to day 21 pi in the TzDM rats. There were no adult worms detected on day 28 pi, but rather, muscle larvae were found in the TzDM rats. There was a significant increase (*p* < 0.001) in the muscle larvae in the TzDM rats from day 28 pi to day 35 pi, with a mean of 16.56 ± 3.58 lpg and 19.20 ± 4.21 lpg, respectively (Figure 1).

### 2.2. Natural Discrimination between TzDM Rats and the Control Rats

**Figure 2:**
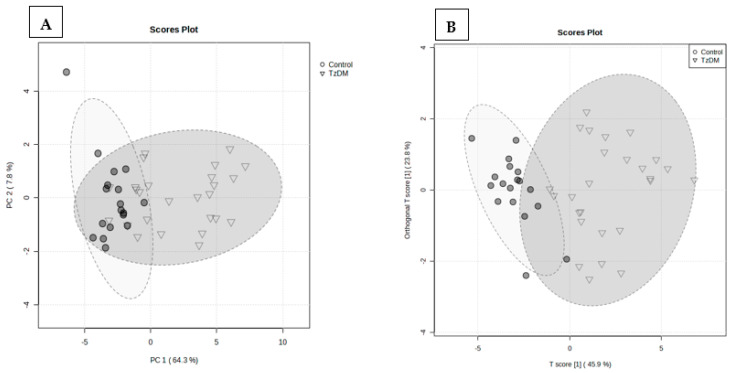
Partial least squares discriminant analysis (PLS-DA) (**A**), and orthogonal partial least squares discriminant analysis (OPLS-DA) score plots (**B**) of control and TzDM groups of rats. The score plots, the abscissa PC1, and the ordinate PC2 represent the scores of the principal components ranking the first and the second, respectively, and different shapes/colors of the scattered points represent the different groups of the samples. The explained variances are shown in brackets. TzDM = type 2 diabetes mellitus-induced rats infected with *Trichinella zimbabwensis*.

A total of 938 metabolites were identified in the serum samples of the control and comorbid (TzDM) group. To explore the metabolic differences between the two groups, the control and comorbid groups, a multivariate statistical analysis was performed on the GCxGC-TOF-MS dataset. Figure 2 shows the variation between the two groups, with a PLS-DA explaining a total variance of 72.1%, of which PC1 was 64.3% and PC2 accounted for 7.8%. An orthogonal partial least squares discriminant analysis (OPLS-DA) was used to build a model (statistical classifier) to differentiate the comorbid and control groups. The OPLS-DA was performed and depicted a modeling parameter of R2X = 97.4% that was indicative of the total explained variation in response Y, and R2Y = 43.2% representing the cross-validated variation accounted for by response Y. As shown in Figure 2, comorbidity caused noticeable changes in the metabolite signature when compared to the control rats.

### 2.3. Metabolite Changes between the Groups

**Figure 3:**
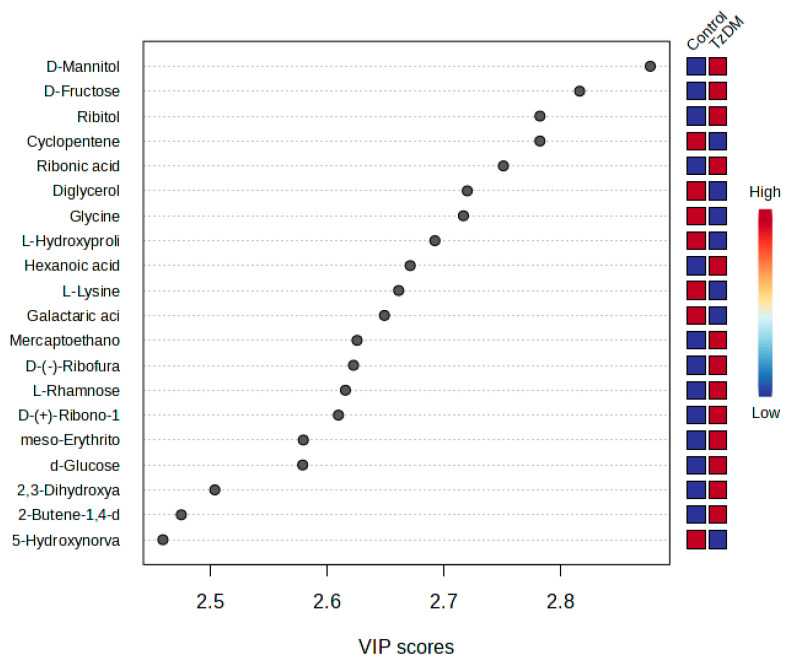
PLS-DA VIP plot. The top 20 differentiating metabolites between control and comorbid (TzDM) groups of rats. TzDM = type 2 diabetes mellitus-induced rats infected with *Trichinella zimbabwensis*.

The metabolites that had significant changes (*p* < 0.05) were identified based on the absolute cut-off values of the correlation coefficient and variable importance in projection (VIP > 1.5), and these were considered differentiating metabolites. A total of 62 potential biomarkers were identified that differentiated the comorbid group (TzDM) from the control rats (Table 1, Figure 3). Metabolites that had a VIP score > 1.5 were interpreted as being highly influential and play an important role in distinguishing the control group from the TzDM group (Table 1).

From the top 20 distinguishing metabolites, carbohydrates were the dominating metabolite group, followed by amino acids. D-mannitol and D-fructose were two carbohydrates that significantly changed between the comorbid and the control groups. D-glucose was upregulated in the comorbid group in comparison to the control group. Ribitol was a carbohydrate from the top 20 metabolites that were downregulated in the comorbid group when compared to the control group (Table 1, Supplementary Table S1). Amino acid metabolites such as L-hydroxyproline, L-lysine, and hydroxy-norvaline were downregulated in the comorbid group when compared to the control group. All organic acids were increased in the comorbid group as compared to the control.

### 2.4. Weekly Metabolite Changes Post-Infection

There was a change in the relative concentration of the identified differentiating metabolites at different stages post-infection (Supplementary Figure S1). However, there was no significant (*p* < 0.05) difference in the relative concentration of the top 20 differentiating metabolites on different days. The relative concentration of the carbohydrates D-mannitol, d-fructose, and d-rib furanose, but not d-glucose and L-rhamnose, increased from day 0 pi to day 21 pi, concurrently with the decrease in adult worms in the intestine. Additionally, these metabolites decreased during day 28 pi and day 35 pi as the muscle larval count increased. Glucose and L-rhamnose increased from day 0 pi to day 21 pi and decreased on day 28 pi. The relative concentration of the amino acid, L-hydroxyproline, increased from day 0 pi to day 21 pi and decreased from day 28 pi to day 35 pi. 

### 2.5. Pathway Discovery and Impact Analyses

**Figure 4:**
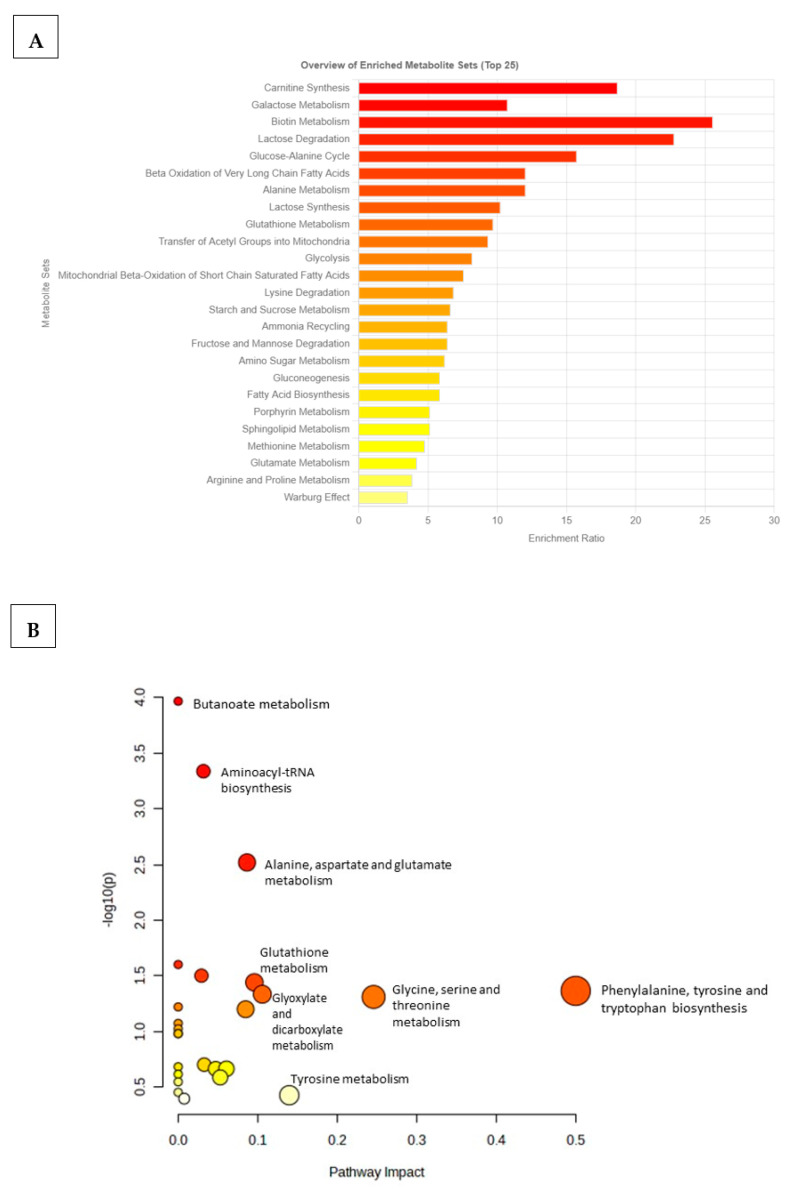
Metabolic pathways associated with the top 20 identified potential metabolites in type 2 diabetes mellitus-induced rats infected with *Trichinella zimbabwensis*. (**A**) The horizontal bar graph shows the most significant metabolite sets identified during analysis. Bar colors are based on *p*-values (lower *p*-values correspond to a darker red), while bar lengths are based on the fold enrichment. (**B**) Metabolomics Pathway Analysis (MetPA) showing matched pathways as circles. The node size is proportional to the enrichment ratio. Light yellow to red indicates the *p*-value from small to large. The color and size of each circle are based on the *p*-value and pathway impact value, respectively. The most impacted pathways with high statistical significance scores are indicated with their names.

To identify which metabolic pathways were most impacted/affected in the comorbid group, an overview of the *p*-values from the impact analysis, topology analysis, and enrichment analysis (MSEA) was performed (Supplementary Table S2). MSEA was essential for determining biologically meaningful patterns that are significantly enriched in quantitative metabolomic data (Figure 4). Based on the top 20 identified differentiating metabolites, biotin metabolism, carnitine synthesis, lactose degradation, galactose metabolism, and glucose–alanine metabolism were metabolic pathways that were significantly affected in the TzDM group (Figure 4A). Additionally, metabolic pathways that were less enriched or changed in the TzDM group included glutamate metabolism, arginine and proline metabolism, and the Warburg effect. The metabolome overview obtained through Metabolic Pathway Analysis (MetPA) showed phenylalanine, tyrosine, and tryptophan biosynthesis metabolism and glycine, serine, and threonine metabolism as the most impacted metabolic pathways in rats with comorbidity (Figure 4B, Supplementary Table S3).

### 2.6. Diagnostic Accuracy 

**Figure 5:**
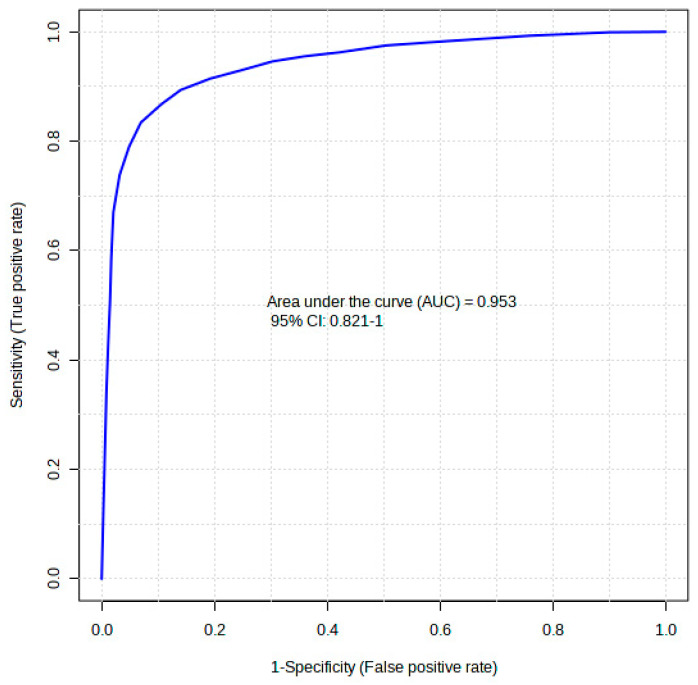
A receiver operating curve (ROC) of the top 20 identified potential biomarkers for distinguishing comorbid group, i.e., type 2 diabetes mellitus-induced rats infected with *Trichinella zimbabwensis*.

The diagnostic performance of each of the 20 differentiating metabolites in discriminating the comorbidity group from the control was evaluated using a receiver operating curve analysis (ROC). In Table 1, the ROC analysis showed that hexanoic acid, diglycerol, and D-fructose had higher diagnostic abilities, with areas under the curve (AUCs) of 0.869, 0.869, and 0.858 (*p* < 0.05), respectively (Table 1). The top 20 identified metabolite markers showed good discriminating power. A combination of the 20 identified metabolites in a multivariate model showed that the AUC obtained for the final model was 95.3 (95% CI 0.82–1) (Figure 5), indicating an excellent discriminative power.

## 3. Discussion

In this study, a metabolic footprint and changes in metabolism during comorbidity were observed, which showed substantial changes in the metabolome. The metabolite groups that were observed to be impacted included carbohydrates and amino acids. Studies conducted on human infection with parasitic worms indicate that worms have a positive effect on metabolic outcomes [22]. Moreso, Berbudi, Ajendra [23] demonstrated that, in both human and animal studies, helminth infection may protect against the establishment of T2DM. The present study aimed to determine the metabolite changes/shifts in T2DM-induced Sprague Dawley rats infected with a tissue-dwelling nematode parasite, *T. zimbabwensis*, and the metabolic pathways that were impacted or perturbed during comorbidity. Additionally, this study was an extension of our previous work that focused on the metabolomics of T2DM in rats compared with a healthy control [24] to further confirm if infection with tissue-dwelling nematodes such as *T. zimbabwensis* ameliorates or attenuates T2DM in rats. Our previous study results showed that the metabolite signature of diabetic rats was different from that of the non-diabetic control group. Additionally, the metabolite changes observed between the diabetic and non-diabetic control group were attributed to the increase in amino acids such as glycine, L-asparagine, and L-serine.

Infecting T2DM rats with *T. zimbabwensis* caused noticeable changes in the number of both adult worms and muscle larvae. Comparing the larval counts in the present study with our previous work on *T. zimbabwensis* [25], the non-diabetic rats infected with *T. zimbabwensis* had a higher number of adult worms compared to rats that had comorbidity (TzDM), indicating that T2DM had an impact on the establishment of adult worms in the hosts and might have resulted in changes in the animal host metabolome. This finding is interesting in that most studies reporting the amelioration of T2DM in hosts with helminth infections involve infections preceding the onset of T2DM. According to Dunn [26], changes in metabolite concentrations result in the perturbation of building blocks for a variety of biochemicals and structures, and recently include the evidence that helminths and their excretory/secretory products regulate the immune responses of the mammalian host and their influence on macrophage and β-cell crosstalk [18]).

In the present study, a distinct separation in the metabolite signature and metabolic pathways between the groups was observed. The metabolic groups that were significantly impacted and changed during comorbidity (TzDM) included carbohydrates and amino acids. Carbohydrates are involved in various processes such as glycemic control, energy metabolism and insulin secretion, lipid metabolism, and colonic function [27]. The results from our study showed that, from the top 20 differentiating metabolites, all carbohydrates were upregulated in the comorbid group, with d-mannitol, ribitol, L-rhamnsose, and d-fructose causing prominent changes compared to the control rats. In our previous work comparing T2DM rats with non-diabetic controls [24], the metabolites that were detected to be common between this study and the aforementioned included d-mannitol, ribitol, d-talofuranose, d-galactose, gluconic acid, diglycerol, pinitol, L-tyrosine, and d-fructose (Figure S2). It can be considered that *T. zimbabwensis*, in comorbidity with T2DM, caused a significant metabolic shift in the carbohydrates, amino acids, organic compounds, and fatty acids.

According to Chukwuma, Matsabisa [28], d-mannitol may show anti-hyperglycemic potential with increased muscle glucose uptake and the inhibition of intestinal glucose absorption. Moreover, Chukwuma, Matsabisa [28] reported that in T2DM rats, an acute administration of d-mannitol inhibited glucose absorption in the small intestines, which was accompanied by reduced blood glucose. In our previous study, the d-mannitol concentration in the T2DM group was higher compared to the control group [24]. However, in the current study, the concentration of d-mannitol was lower in the comorbid group compared to our previous findings on the T2DM group. During *T. zimbabwensis* infection, adult worms were found in the intestines of the host for a short period of time before they were ejected by the host after producing a huge number of larvae, which penetrate the intestinal mucosa and migrate to the striated muscles [24]. According to Tielens [29] and Chappell and Chappell [30], helminths’ adult parasite stages are dependent on carbohydrates, which are obtained from their host, for their energy metabolism. Therefore, we can speculate that the decrease in d-mannitol during comorbidity in the current study was due to a high intake by the adult worms during their ontogeny. The site of predilection of *T. zimbabwensis* larvae is striated muscles [31], where they cause dramatic changes in muscle architecture. Therefore, the decrease in the concentration of d-mannitol and d-fructose observed in our current study might have been attributed to the increase in the muscle larvae that was observed in the comorbid group from 21 days post-infection to 35 days post-infection. This decrease in these metabolites can be due to the competition between the host and the parasite that uses these for growth and as an energy source [32].

D-fructose and d-mannitol were involved in four metabolic pathways, which included starch and sucrose metabolism, fructose and mannose degradation, amino sugar, and galactose metabolism. The decrease in these metabolites resulted in the downregulation of these metabolic pathways, since d-fructose is an intermediate of these pathways. According to Ang and Yu [33], d-fructose in its pure form can be a determining factor that can cause T2DM and various other metabolic disorders. More so, avoiding excessive intake of fructose may decrease propensity for T2DM, and in human and animal studies, d-fructose has been reported to cause insulin resistance [34,35]. The conversion of d-fructose to glucose increases the risks of T2DM. Kolderup and Svihus [36] reported that d-fructose can contribute negatively to blood glucose homeostasis by resulting in insulin resistance in the liver. A study by Silas and Tshilwane [17] found that rats that had a comorbidity of diabetes and *T. zimbabwensis* infection had an increased insulin level on day 35 post-infection when compared to the diabetic group. Additionally, the observed increase was said to be via the insulin signaling pathway. Therefore, *T. zimbabwensis* infection had a positive impact as it caused a reduction in the concentration of d-fructose during comorbidity as compared to rats that were only diabetic.

Emerging evidence suggests that amino acids may play a crucial role in the prevention of diabetes and diabetes-associated complications [37]. According to Zhou and Sun [38], an increase in circulating amino acids is associated with the onset of diabetes. In this study, the amino acids that were detected included L-tyrosine, glycine, diglycerol, L-lysine, and L-hydroxyproline, and their concentration was detected to be increased in our previous study [24], in the T2DM group when compared to the non-diabetic control group and TzDM group. In our previous work, the concentration of L-tyrosine was high in the T2DM group [24] when compared to the control and, in the current work, on the TzDM groups. Previous studies have reported that insulin resistance is related to the metabolism of L-tyrosine [39]. Moreover, according to Koeck and Corbett [39], increased levels of tyrosine might inhibit insulin signaling, which is associated with the development of T2DM. Tinker and Sarto [40] reported that increased amino acid levels are associated with increased insulin resistance and increased risk of T2DM. Therefore, it can be confirmed that *T. zimbabwensis* infection had a significant role in decreasing amino acid metabolism in the TzDM group and, therefore, reducing the chances of insulin resistance and risks of T2DM.

L-lysine was among the common metabolites between this current work and our previous work [24], where its relative concentration was higher in the comorbid group compared to the diabetic and control group. L-lysine is an amino acid that is essential for calcium absorption, the production of body hormones and enzymes, and the building of muscle protein [41]. According to Ren and Rajendran [42], pathogens are capable of modulating amino acids for their own benefits. In animal studies, lysine has shown beneficial effects in treating/preventing diabetes and its complications such as retinopathy, nephropathy, and neuropathy [43]. Based on the function of L-lysine, this suggests that increased levels of L-lysine had a positive impact during the comorbidity of *Trichinella* and diabetes, and this is supported by Jozi and Kheiripour [44], who found that L-lysine ameliorated the progression of diabetic nephropathy in rats. According to Jafarnejad and Bathaie [45], lysine was beneficial in diabetes-induced model rats where it caused a reduction in blood glucose levels and acted as an inhibitor of protein glycation. L-Lysine was found to be associated with the biotin metabolism pathway, which is essential for gluconeogenesis, amino acid catabolism, and fatty acid synthesis [46]. In humans, L-lysine reacts with glucose and attenuates the glucose response to ingested glucose without a change in insulin response [47].

In our previous study, we found that glucose concentration was higher in the diabetic group; however, the glucose level decreased when *T. zimbabwensis* was introduced to the diabetic rats. A study by Onkoba and Chimbari [48] reported a decrease in blood glucose and serum insulin in mice infected with *T. zimbabwensis* compared to non-infected control mice. A study by Thabet, Saleh [49] on the effect of schistosomiasis on glucose uptake via the diaphragm found that the helminth *S. mansoni* caused a decrease in glucose uptake in diabetic rats compared to non-diabetic control mice. Moreover, Taira, Yazawa [16] found that the parasite *Syphacia muris* caused a decrease in blood glucose levels in diabetic rats compared to the control. Our results are in accordance with previously conducted studies on helminths and diabetes, as we found that *T. zimbabwensis* infection influenced glucose metabolism and decreased glucose concentration during comorbidity.

Other metabolic pathways that were significantly impacted during comorbidity included lactose degradation, fatty acid biosynthesis, phenylalanine metabolism, butanoate metabolism, aminoacyl -tRNA biosynthesis, the glucose–alanine cycle, and biotin metabolism. Zhou and Sun [38] reported that an increase in phenylalanine was a cause of T2DM in C57BL/6J mice, as these showed an increase in fasting blood glucose, reduced glucose tolerance, and reduced insulin tolerance. The metabolites that were associated with metabolic pathways included L-tyrosine, which was higher in the TzDM group compared with the control and previous diabetic group. D-glucose and d-galactose were metabolites involved in lactose degradation. Both metabolites were downregulated in the TzDM group when compared to the T2DM group in our previous study [24].

This study found other important metabolites that were significant and impacted/changed due to the comorbidity of diabetes and *T. zimbabwensis*. These metabolites included oleic acid, arsenic acid, aucubin, d-pinitol, L-glutamic acid, and gluconic acid. Oleic acid is a monounsaturated fatty acid (MUFA) and is present in both plants and animals. Furthermore, clinical trials and prospective studies reported that a high intake of oleic acids can improve metabolic syndromes and cardiovascular risk factors associated with T2DM [50]. In the glucose-sensitive INS-1 cell lines, oleic acid increases insulin secretion [51]. The concentration of oleic acid in our current study was higher in the comorbid group compared to the control group. Oleic acid is found to be effective in reversing the inhibitory effect in insulin production of inflammatory cytokines [51]. The increase that we observed during comorbidity can be attributed to oleic acid improving and ameliorating diabetes.

Gluconic acid was also an important metabolite that was found to have decreased in the comorbid study when compared to the diabetes group. According to [52], gluconic acid is formed enzymatically from glucose and is associated with hyperglycemia. The decrease in gluconic acid can be attributed to the observed decrease in glucose. The other metabolite detected to have affected insulin resistance was glutamic acid. Glutamic acid levels decreased in the comorbid group when compared to the diabetic group and, according to Samocha-Bonet and Chisholm [53], glutamine reduces postprandial glycemia.

## 4. Materials and Methods

### 4.1. Study Design

Forty-five male Sprague Dawley (SD) rats with a body weight between 160 g and 180 g were used, and these were randomly selected from a colony that was bred and maintained at the Biomedical Resource Unit (BRU), University of KwaZulu-Natal (Westville campus), Durban, South Africa. All experimental protocols and procedures of the study were reviewed and approved by the animal research ethics committee of the University of KwaZulu-Natal under the reference number AREC/028/018D.

**Figure 6:**
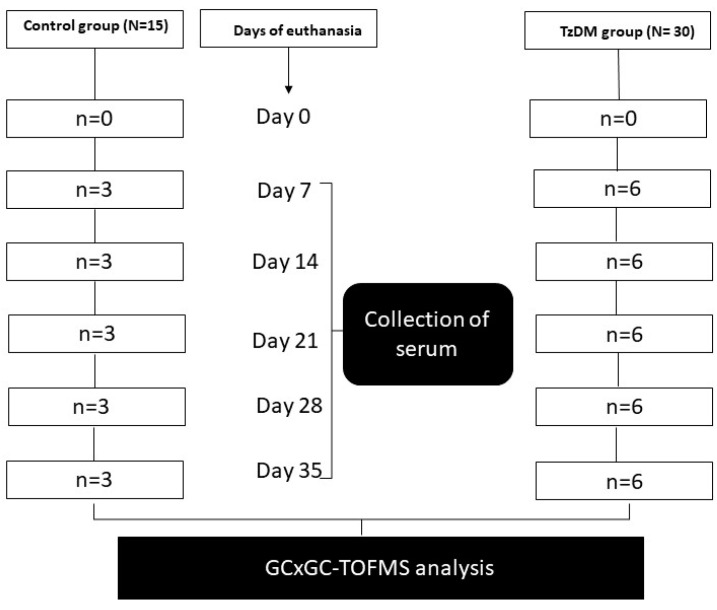
Experimental design.

The experimental animals were maintained and subjected to laboratory conditions with a constant room temperature (22 ± 2 °C), illumination in a 12 h light/dark cycle, and a carbon dioxide content of <5000 ppm. They were given free access to drinking water and fed ad libitum a standard rat chow diet from Meadows, Pietermaritzburg, South Africa, during the entire experimental period. The experimental animals were acclimatized for one week in their new cage environment and randomly assigned to two groups, namely the non-diabetic control group (n = 15) and the T2DM-induced and *T. zimbabwensis* comorbid group (TzDM) (n = 30) (Figure 1). Six animals from the comorbidity group and three from the control group were euthanized using isofor inhalation in a gas chamber at days 7, 14, 28, and 35 post-infection (Figure 6). On each day of euthanasia, blood samples were collected from the rats via a cardiac puncture to obtain sera. The serum samples were analyzed using an untargeted metabolomics approach with two-dimensional gas chromatographic time-of-flight mass spectrometry (GCxGC-TOF/MS).

### 4.2. Type 2 Diabetes Mellitus Induction

The induction of T2DM in the experimental animals was achieved as described in [54]. In brief, animals in the TzDM group were orally administered 10% fructose solution ad libitum for two weeks before the administration of streptozotocin (STZ) (Sigma, St. Louis, MO, USA) to induce insulin resistance. After 14 days of taking the fructose solution, the rats were fasted overnight, and streptozotocin (STZ) (Sigma, St. Louis, MO, USA) was administered intraperitoneally at a dose of 40 mg/kg bw. The STZ solution used was first freshly dissolved in a citrate buffer (pH 4.5) before being administered. A glucometer (Glucoplus Inc., Saint-Laurent, QC, Canada) was used to measure the non-fasting blood glucose level (NFBG) from the tail vein. Animals that had NFBG levels of >18 mmol/L were designated as diabetic, and those with levels <18 mmol/L were considered non-diabetic and were excluded from the study. Two days after the administration of STZ, the rats were found to be diabetic. Their glucose levels were measured every two days throughout the experimental period. Animals in the control group were given water ad libitum.

### 4.3. Trichinella zimbabwensis Infection

The experimental animals were infected with *T. zimbabwensis* using a crocodile-derived *T. zimbabwensis* (ISS1209) strain maintained in Sprague Dawley stock rats at the Biomedical Research Unit, University of KwaZulu-Natal, South Africa, Durban. From the stock rats, muscle larvae were obtained from the digestion of carcasses following the method described by Kapel and Gamble (2000). Seven days post-induction of T2DM, the experimental animals in the comorbidity (TzDM) group were infected with *T. zimbabwensis* larvae via oral gavage at a dosage of 3 mL/g of rat body weight using an 18 G curved oral dosing needle.

### 4.4. Trichinella zimbabwensis Worm Recovery

Adult worm recovery from the intestines was conducted using the protocol described by [55] at days 7, 14, and 21 post-infection. *Trichinella zimbabwensis* adult parasites were viewed and counted using a Zeiss Stemi DV4 stereo microscope (Jena, Germany). To detect and confirm parasite larvae, animal muscle tissues were digested following the modified artificial digestion protocol as described by Pozio [31] at days 28 and 35 post-infection.

### 4.5. Analysis of Serum Samples

Blood samples were collected from the control and TzDM groups via cardiac punctures at days 7, 14, 21, 28, and 35. A total of 45 serum samples (2 mL aliquot each) were collected from individual animals for the control and TzDM groups and then stored at −80 °C in a Bio Ultra freezer (Snijders Scientific, Tilburg, The Netherlands) until they were transported to the North-West University (Potchefstroom, South Africa) Center for Human Metabolomics for analysis using GCxGC-TOF/MS.

### 4.6. Untargeted GCxGC-TOFMS Approach

For the GCxGC-TOFMS analysis, a Pegasus GCxGC-TOFMS (Leco Corporation Joseph, St. Joseph, MI, USA) that uses an Agilent 7890A GC (Atlanta, GA) coupled to a time-of-flight mass spectrometer (TOFMS) (Leco Corporation, St. Joseph, MI, USA) equipped with a Gerstel Multipurpose sampler was used for the chromatographic analyses of the derivatized samples. One µL of serum extract was randomly injected at a split ratio of 1:50, and the carrier gas used was helium at a flow rate of 1 mL/min. For the entire run, the temperature of the injectors was kept constant at 270 °C. A Restek Rxi-5Sil MS capillary column (29.145 m × 0.25 μm d.f.) was used as the primary column. The primary oven was programmed to an initial temperature of 70 °C for 2 min to achieve compound separation. Subsequently, this was followed by a 4 °C per minute increase to a final temperature of 300 °C, where it was maintained for 2 min. The second separation of compounds was achieved using a Restek Rxi-17 (1.400 m, 0.25 µm i.d., 0.25 μm d.f.) column. The secondary column used was set to the same temperature parameters as those of the primary column. The filament bias was EI at 70 eV, while the detector voltage was at 1600 V. Subsequently, the mass spectra were collected at an acquisition rate of 200 spectra per second, with a source temperature of 220 °C and a solvent delay of 400 s from 50 to 800 *m*/*z* [56].

### 4.7. Peak Identification

The Leco Corporation Chroma-TOF software (version 4.50) was used for peak finding and mass spectral deconvolution at an S/N ratio of 100, with a minimum of three apexing peaks. Using the mass fragmentation patterns generated via the MS, together with their respective GC retention times, the identities of these peaks were determined by comparing them to commercially available NIST spectral libraries (mainlib, replib).

### 4.8. Data Clean-Up

A data clean-up was conducted using Microsoft Excel to remove features that were not reliably measured. The reliability of each variable was assessed by calculating the relative standard deviation (standard deviation divided by the mean) across all quality control samples of the TzDM and control samples. Features with a relative standard deviation above 50% were excluded from further analysis. This was accomplished by replacing all zero values with half the minimum observed value for the dataset, which served as an estimate for the detection limit. The data were log-transformed and scaled using MetaboAnalyst, where the data were not normally distributed. A natural shifted log transformation was performed to correct the skewness distribution of the variables, followed by auto-scaling to place all variables to obtain normality [57].

### 4.9. Identification of Compounds and Pathway Analysis

The potential metabolite biomarkers were selected based on the greatest variable importance in projection (VIP) values and had to be statistically significant (VIP > 1.5 and *p* < 0.05) [57,58]. The identified metabolic biomarkers were mapped onto the Kyoto Encyclopedia of Genes and Genomes (KEGG) pathway network using iPath 3.0 (http://pathways.embl.de (Accessed 20 July 2023), a web-based application used to visualize and analyze cellular pathways. A metabolite set enrichment overview (MSEA) was constructed using the significant metabolites to elucidate the pathways that these significant metabolites are involved in using MetaboAnalyst version 5.0 (https://www.metaboanalyst.ca/docs/Publications.xhtml (Accessed 20 July 2023)), an online tool for analyzing and interpreting metabolic enrichment. Moreover, MetPA was incorporated with MetaboAnalyst for pathway analyses.

### 4.10. Statistical Analysis

Multivariate, partial least squares discriminant analyses (PLS-DAs) were applied for statistical analysis using a web-based server, MetaboAnalyst version 5.0. A graphical representation of data was constructed using the Prism software, version 5. The integral intensity of the metabolites was presented as the median with the inter-quartile range (IQR). The data were normalized using auto-scaling and log transformation. For data overview and pattern discovery, a PCA was first constructed followed by a supervised classification method, PLS-DA. The PLS-DA was used to determine the group membership of individual samples, based on their metabolic profiles. Thereafter, a supervised orthogonal partial least squares discriminant analysis (OPLS-DA) method was performed to improve the separation between the group of samples and to minimize other biological analytical variations. The OPLS-DA was cross-validated via permutation (n = 20). Thereafter, the goodness-of-fit parameters were calculated (R2X, R2Y, and Q2Y) using MetaboAnalyst 5.0. The receiver operating curve analysis (ROC) was used to further evaluate the predictive ability of the identified potential metabolic biomarkers. Moreover, the area under the curve was used to determine the diagnostic accuracy of the biomarkers, where 0.8 < AUC < 0.9 was considered good and 0.9 < AUC ≤ 1.0 was considered excellent. To assess if there was a significant difference (*p* < 0.05) in the levels of potential biomarker metabolites at different days, a one-way analysis of variance (ANOVA) was performed, followed by post hoc analyses using Fisher’s LSD. For quality assurance, aliquots from the pooled QC (quality control) samples were used to determine if the samples were consistently analyzed within and across batches. A PCA score plot was used to ensure the absence of possible batch effects in the generated data.

## 5. Conclusions

We conclude from this study that infecting diabetic rats with a tissue-dwelling nematode, *T. zimbabwensis*, originates a shift in the metabolome, causing significant changes in metabolic pathways. The nematode infection caused a downregulation of glucose and glucose-producing pathways in diabetic rats. Moreover, the nematode parasite establishment in the host was impacted in the diabetic rats, where the adult and larval stages were reduced. The results depict that a tissue-dwelling nematode such as *T. zimbabwensis* has a protective role against the progression of T2DM and its possible complications in the host. Hence, the application of metabolomics (untargeted) proved useful as a tool for studying metabolic shifts during comorbidity, as it unraveled a separation between the comorbid and control groups. We recommend future studies using a targeted metabolomics approach to further study the identified differentiating metabolites. Moreover, the use of *T. zimbabwensis* or other tissue-dwelling helminth species as a model for studying T2DM comorbidity with other pathogens in laboratory animal-based studies is recommended.

## Figures and Tables

**Table 1:** Top 20 differentiating metabolites with diagnostic accuracy, fold change, and metabolite group in the type 2 diabetes mellitus-induced rats infected with *Trichinella zimbabwensis*.

Metabolite	Group	AUC	VIP	Log2 FC	Variation
D-Mannitol	Carbohydrate	0.837	2.877	1.326	UP
D-Fructose	Carbohydrate	0.858	2.8165	1.0025	Up
Ribitol	Carbohydrate	0.846	2.7825	−0.773	Down
Cyclopentene	Xenobiotic	0.863	2.7825	0.620	Up
Ribonic acid	Organic compound	0.861	2.7512		
Diglycerol	Amino acid	0.869	2.7203	0.966	Up
Glycine	Amino acid	0.837	2.717	0.437	Up
L-Hydroxyproline	Amino acid	0.850	2.6925	−2.976	Down
Hexanoic acid	Fatty acid	0.869	2.6714	1.0446	Up
L-Lysine	Amino acid	0.829	2.6615	0.618	Down
Galactaric acid	Organic compound	0.850	2.6493	0.735	Up
Mercaptoethanol	Xenobiotic	0.825	2.6259	1.437	Down
D-(−)-Rib furanose	Carbohydrate	0.835	2.6228	1.165	Up
L-Rhamnose	Carbohydrate	0.826	2.6159	0.851	Up
D-(+)-Ribono-1,4-lactone	Organic compound	0.856	2.6099	1.098	
meso-Erythritol	Organic compound	0.831	2.5799	0.262	Up
d-Glucose	Carbohydrate	0.835	2.5793	0.966	Up
2,3-Dihydroxyacrylic-Acid	Organic compound	0.8365	2.504	0.652	Up
2-Butene-1,4-diol	Xenobiotic	0.826	2.4753	0.876	Up
5-Hydroxynorvaline	Amino acids	0.825	2.877	0.784	Down

FC—old change, VIP—variable importance in projection, AUC—area under the curve (the measure of the ability of a binary classifier to distinguish between classes).

## Data Availability

Data are available upon request.

## Supplementary Materials

The following supporting information can be downloaded at https://www.mdpi.com/article/10.3390/ijms242417211/s1.
