# Supplementary material for: Metabolomics (Non-Targeted) of Induced Type 2 Diabetic Sprague Dawley Rats Comorbid with a Tissue-Dwelling Nematode Parasite
Source: Int J Mol Sci. 2023 Dec 7;24(24):17211. doi: 10.3390/ijms242417211 (PMC10743009; doi:10.3390/ijms242417211)
Supplement: Supplementary file 1 [file ijms-24-17211-s001.zip › ijms-2686360-SM.pdf]

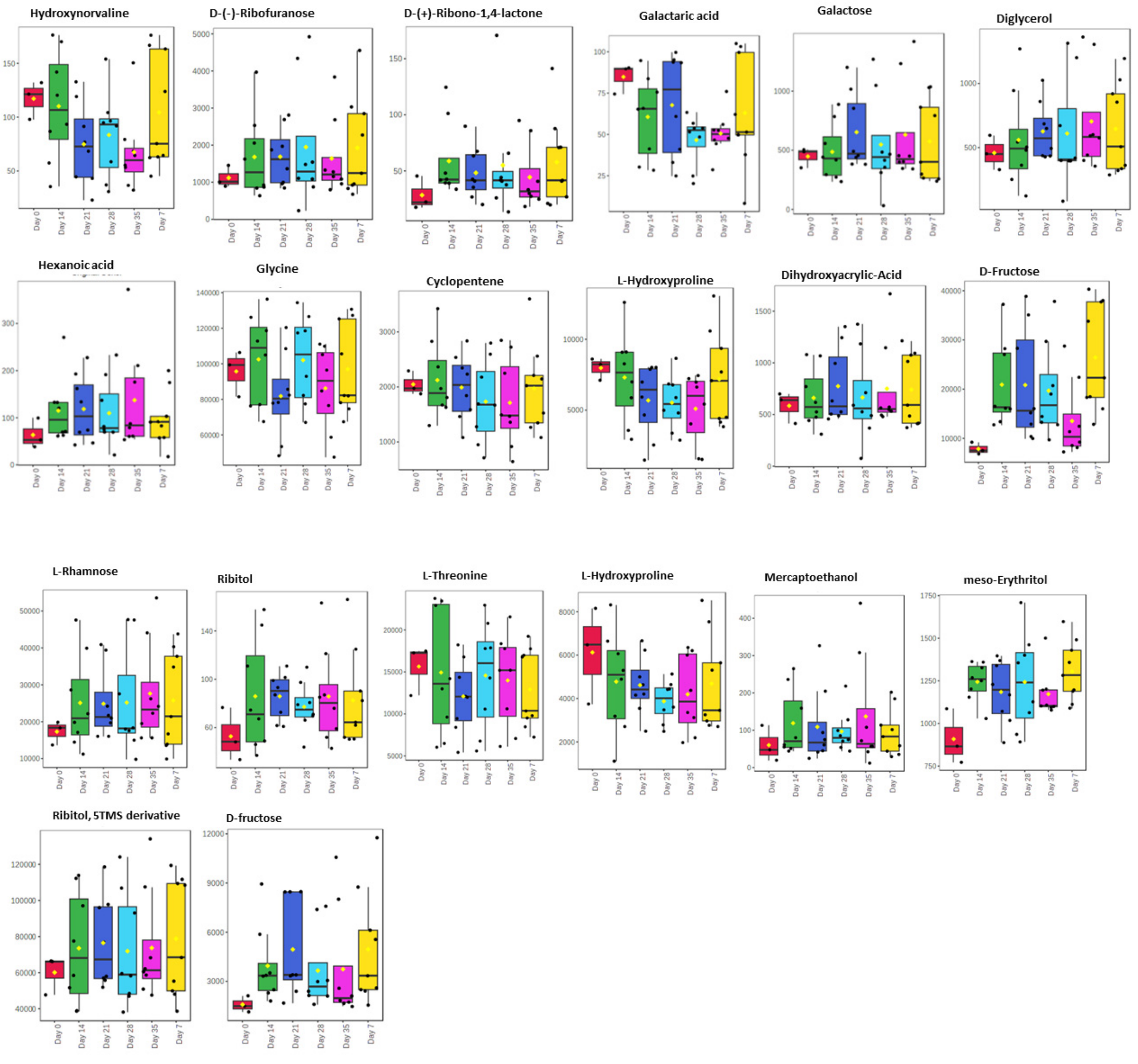

Figure S1. Relative concentration of the top 20 potential biomakers for diabetic SD-rats infected with *Trichinella zimbabwensis metabolite* at different days post infection.

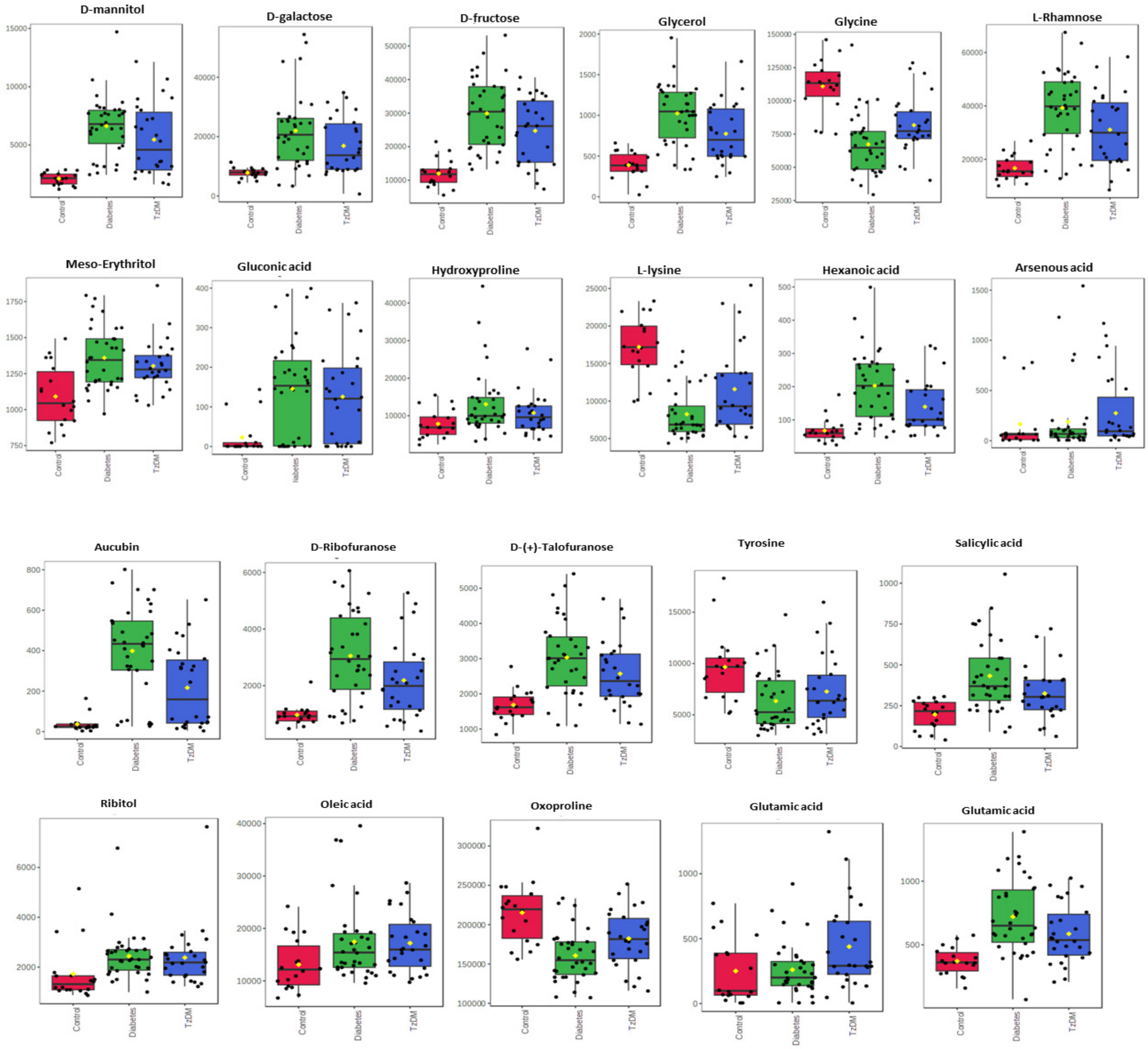

Figure S2. Relative concentration of metabolites that were common between the current study and our previous study on diabetes and control groups control (red), diabetes (T2DM-green), and comorbidity group (TzDM- blue).

Table S1. Illustration of potential biomarkers of diabetic SD-rats infected with *Trichinella zimbabwensis* and their PLS-DA VIP- values.

**Metabolites**	**VIP**	**Matabolites**	**VIP**
**D-Mannitol**	2.877	Oxoproline	2.134
D-Fructose	2.8165	Glycine	2.0828
Ribitol	2.7825	Pentitol	2.072
Cyclopentene	2.7825	Butylcatechol	2.0408
Ribonic acid	2.7512	hydroxypropionic acid	2.0358
L-Hydroxyproline	2.7203	Boric acid	2.0356
Glycine	2.717	L- (+)-Threose	2.0187
L-Hydroxyproline	2.6925	Diethylamine	2.0128
Hexanoic acid	2.6714	3-Hydroxybutyric acid	2.0012
L-Lysine	2.6615	acetate	1.9864
Galactaric acid	2.6493	D-Mannitol, 6TMS derivative UM 205	1.9839
Mercaptoethanol	2.6259	N-Ethyldiethanolamine,	1.9766
D-(-)-Ribofuranose	2.6228	N-Acetylaspartic-Acid	1.9742
L-Rhamnose	2.6159	Salicylic acid	1.9727
D-(+)-Ribono-1,4-lactone	2.6099	2-Hydroxycyclohexane-1-carboxylic acid	1.9725
meso-Erythritol	2.5799	Cyclopropanecarboxylic acid	1.9699
d-Glucose	2.5793	d-Glucose, 2,3,4,5,6-pentakis	1.9697
2,3-Dihydroxyacrylic-Acid	2.504	4-Aminobutanoic acid	1.9662
2-Butene-1,4-diol	2.4753	á-Hydroxypyruvic acid	1.9652
5-Hydroxynorvaline	2.4595	Tyrosine	1.9593
Butanoic acid	2.4215	Succinic acid	1.9592
Butanoic acid, 2,2-dimethy	2.3746	oxobutyric acid	1.9513
D-Pinitol	2.3641	L-Asparagine	1.9306
1-Methoxy-2-propanol	2.3379	Methyl stearate	1.9218
D- (+)-Talofuranose	2.3306	Acetic acid	1.9154
Ribitol	2.3187	Isonicotinic Acid	1.9061
d-Glucose, 2,3,4,5,6-pentakis-O-(trimethylsilyl)-	2.2811	Gluconic acid	1.9015
D- (-)-Ribofuranose	2.2282	3-Pyridinol	1.9011
D-Galactose	2.216	L-Threonine	1.8972
Eicosadienoic acid	2.2105	Pentanedioic acid	1.8942
Ribitol, 5TMS derivative	2.1507	Allocholic acid	1.8938

Table S2. MSEA metabolite match for metabolites that separates the three groups.

Metabolites	Match	HMDB	PubChem	KEGG
D-Mannitol	Mannitol	HMDB0000765	6251	C00392
D-Fructose	D-Fructose	HMDB0000660	439709	C02336
Ribitol	Ribitol	HMDB0000508	NA	C00474
Cyclopentene	NA	NA	NA	NA
Ribonic acid	Ribonic acid	HMDB0000867	5460677	C01685
L-Hydroxyproline	4-Hydroxyproline	HMDB0000725	5810	C01157
Glycine	Glycine	HMDB0000123	750	C00037
Hexanoic acid	Caproic acid	HMDB0000535	8892	C01585
L-Lysine	L-Lysine	HMDB0000182	5962	C00047
Galactaric acid	Galactaric acid	HMDB0000639	3037582	C00879
Mercaptoethanol	NA	NA	NA	NA
D-(-)-Ribofuranose	NA	NA	NA	NA
L-Rhamnose	Rhamnose	HMDB0000849	25310	C00507
D-(+)-Ribono-1,4-lactone	NA	NA	NA	NA
meso-Erythritol	Erythritol	HMDB0002994	222285	C00503
d-Glucose	D-Glucose	HMDB0000122	5793	C00221
2,3-Dihydroxyacrylic-Acid	NA	NA	NA	NA
2-Butene-1,4-diol	NA	NA	NA	NA
5-Hydroxynorvaline	NA	NA	NA	NA
Butanoic acid	Butyric acid	HMDB0000039	264	C00246
Butanoic acid, 2,2-dimethy	NA	NA	NA	NA
D-Pinitol	D-Pinitol	HMDB0034219	0	C03844
1-Methoxy-2-propanol	NA	NA	NA	NA
D-(+)-Talofuranose	NA	NA	NA	NA
d-Glucose, 2,3,4,5,6-pentakis-O-(trimethylsilyl)	NA	NA	NA	NA
D-Galactose	D-Galactose	HMDB0000143	439357	C00984
Eicosadienoic acid	Eicosadienoic acid	HMDB0005060	6439848	C16525
Ribitol, 5TMS derivative	NA	NA	NA	NA
Oxoproline	Pyroglutamic acid	HMDB0000267	7405	C01879
Pentitol	Ribitol	HMDB0000508	NA	C00474
Butylcatechol	NA	NA	NA	NA
hydroxypropionic acid	Hydroxy propionic acid	HMDB0000700	68152	C01013
Boric acid	NA	NA	NA	NA
L- (+)-Threose	NA	NA	NA	NA
Diethylamine	Diethylamine	HMDB0041878	8021	C14422
3-Hydroxybutyric acid	3-Hydroxybutyric acid	HMDB0000357	441	C01089
acetate	Acetic acid	HMDB0000042	176	C00033
D-Mannitol, 6TMS derivative UM 205	NA	NA	NA	NA

N-Ethyldiethanolamine	NA	NA	NA	NA
N-Acetylaspartic-Acid	NA	NA	NA	NA
Salicylic acid	Salicylic acid	HMDB0001895	338	C00805
2-Hydroxycyclohexane-1-carboxylic acid	NA	NA	NA	NA
Cyclopropanecarboxylic acid	NA	NA	NA	NA
d-Glucose, 2,3,4,5,6-pentakis	NA	NA	NA	NA
4-Aminobutanoic acid	Gamma-Aminobutyric acid	HMDB0000112	223130	C00334
Ã¡-Hydroxypyruvic acid	NA	NA	NA	NA
Tyrosine	L-Tyrosine	HMDB0000158	6057	C00082
Succinic acid	Succinic acid	HMDB0000254	1110	C00042
oxobutyric acid	NA	NA	NA	NA
L-Asparagine	L-Asparagine	HMDB0000168	6267	C00152
Methyl stearate	Methyl stearate	HMDB0034154	8201	NA
Acetic acid	Acetic acid	HMDB0000042	176	C00033
Isonicotinic Acid	Isonicotinic acid	HMDB0060665	5922	C07446
Gluconic acid	Gluconic acid	HMDB0000625	10690	C00257
3-Pyridinol	NA	NA	NA	NA
L-Threonine	L-Threonine	HMDB0000167	6288	C00188
Pentanedioic acid	Glutaric acid	HMDB0000661	743	C00489
Allocholic acid	Allocholic acid	HMDB0000505	53477699	C17737

Table S3. MetPath table for metabolites impact on each pathway for the control and comorbid group.

Pathway Name	Metabolites Involved	p-Value	FDR	Impact
Butanoate metabolism	(R)-3-Hydroxybutanoate, 4-Aminobutanoate, Butanoic acid, Succinate	1.6E-05	0.001	0.032
Aminoacyl-tRNA biosynthesis	L-Asparagine, Glycine, L-Lysine, L-Threonine, L-Tyrosine	1.5E-04	0.006	<0.001
Alanine, aspartate, and glutamate metabolism	L-Asparagine,4-Aminobutanoate, Succinate	3.6E-03	0.101	0.087
Propanoate metabolism	Succinate,3-Hydroxypropanoate	2.8E-02	0.477	<0.001
Glycolysis / Gluconeogenesis	beta-D-Glucose, Acetate	3.5E-02	0.477	0.029
Glutathione metabolism	Glycine,5-Oxoproline	4.0E-02	0.477	0.096
Phenylalanine, tyrosine, and tryptophan biosynthesis	L-Tyrosine	4.6E-02	0.477	0.500
Glyoxylate and dicarboxylate metabolism	Glycine, Acetate	5.1E-02	0.477	0.106
Glycine, serine, and threonine metabolism	Glycine, L-Threonine	5.4E-02	0.477	0.246
Synthesis and degradation of ketone bodies	(R)-3-Hydroxybutanoate	5.7E-02	0.477	<0.001
Amino sugar and nucleotide sugar metabolism	alpha-D-Galactose, beta-D-Fructose	6.7E-02	0.490	<0.001
Arginine and proline metabolism	4-Aminobutanoate, Hydroxyproline	7.0E-02	0.490	0.085
Valine, leucine, and isoleucine biosynthesis	L-Threonine	8.9E-02	0.578	<0.001
Ubiquinone and other terpenoid-quinone biosynthesis	L-Tyrosine	1.0E-01	0.580	<0.001
Phenylalanine metabolism	L-Tyrosine	1.1E-01	0.580	<0.001
Biotin metabolism	L-Lysine	1.1E-01	0.580	<0.001
Citrate cycle (TCA cycle)	Succinate	2.1E-01	0.958	0.033
beta-Alanine metabolism	3-Hydroxypropanoate	2.2E-01	0.958	<0.001
Pentose phosphate pathway	D-Gluconic acid	2.3E-01	0.958	0.047
Pyruvate metabolism	Acetate	2.3E-01	0.958	0.061
